# Term gravid uterus in a congenital umbilical hernia: a case report

**DOI:** 10.1186/s13256-021-02760-2

**Published:** 2021-04-03

**Authors:** Friday Saidi, Bakari Rajab, Lameck Chinula, Nomsa Kafumba, Maganizo Chagomerana, Jennifer H. Tang

**Affiliations:** 1 University of North Carolina Project-Malawi, P. Bag A-104, Lilongwe, Malawi; 2 Department of Obstetrics and Gynecology, Baylor College of Medicine, Houston, USA; 3 Department of Obstetrics and Gynecology, Kamuzu Central Hospital, Lilongwe, Malawi; 4 Department of Obstetrics and Gynecology, University of North Carolina, Chapel Hill, NC USA

**Keywords:** Congenital umbilical hernia, Pregnancy, Herniorrhaphy

## Abstract

**Background:**

Umbilical hernias are a frequent and well-known pathology in children or adults. Congenital umbilical hernias are commonly diagnosed in childhood, and in adulthood such a hernia is usually acquired. Umbilical hernia in pregnancy may result in serious obstetric complications including antepartum hemorrhage, intrauterine fetal demise, and preterm labor, particularly if incarcerated.

**Case presentation:**

We present a rare case of a congenital umbilical hernia in a term pregnancy. The patient was a 34-year-old African (Malawian) woman, living with human immunodeficiency virus (HIV) and on antiretroviral treatment, gravida 4, with three previous vaginal deliveries, and with two babies weighing 4 kg at birth. We performed herniorrhaphy at caesarean section, and at 3 months of follow-up she had no evidence of a recurrent hernia.

**Conclusion:**

Congenital umbilical hernias are commonly diagnosed in childhood but might first be seen by medical practitioners in adulthood. A patient-centered approach addressing patient complaints, associated risk factors, and possible complications is recommended. Primary repair at caesarean section is a feasible option.

## Background

Umbilical hernias are a frequent and well-known pathology in children and adults. Congenital umbilical hernias are commonly diagnosed in childhood, and in adulthood such a hernia is usually acquired. Umbilical hernias in pregnancy may result in serious obstetric complications including antepartum hemorrhage, intrauterine fetal demise, and preterm labor, particularly if incarcerated [1–3]. We report a rare case of a congenital umbilical hernia in a term pregnancy for which herniorrhaphy was performed at the time of cesarean section, with a positive outcome.

## Case presentation

A 34-year-old African (Malawian) woman, gravida 4, para 3, living with human immunodeficiency virus (HIV) and on antiretroviral treatment, presented to the antenatal clinic at 31 weeks gestation with an umbilical hernia that was noted since birth. She presented with a 3-day history of fever, generalized body pain, and 4-week history of a dragging sensation in the abdomen and episodes of abdominal discomfort.

She had three previous spontaneous vaginal deliveries with the previous two pregnancies, birth weight of 4.3 kg and 4.4 kg, with no prior screening for gestational diabetes. All three previous pregnancies were herniated pregnancies with no notable obstetric complications. The past gynecological and surgical history were unremarkable.

**Fig. 1:**
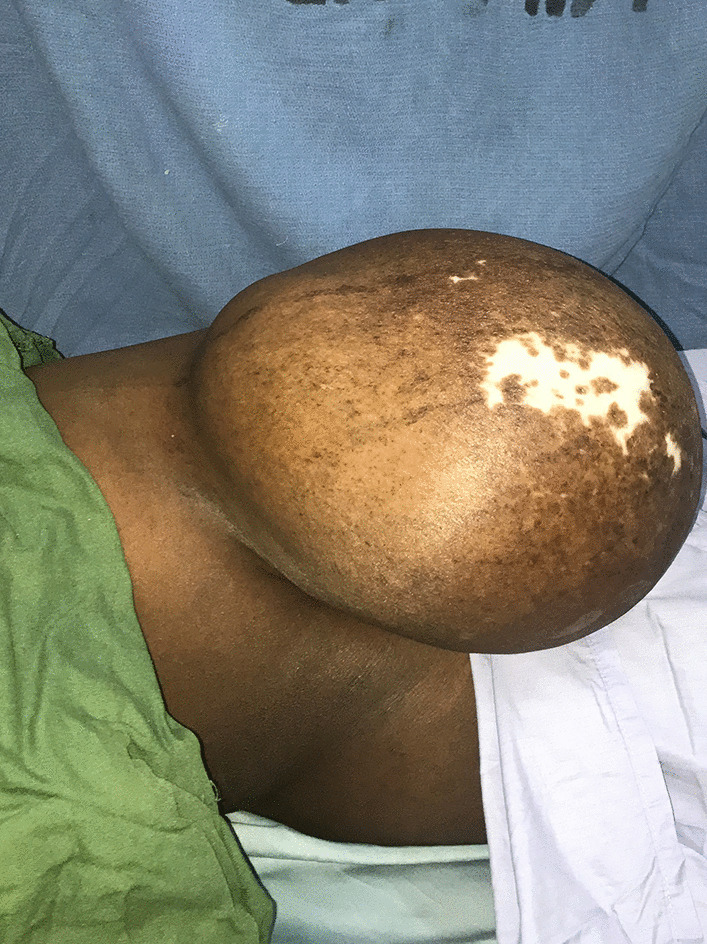
Herniated term pregnancy

**Fig. 2:**
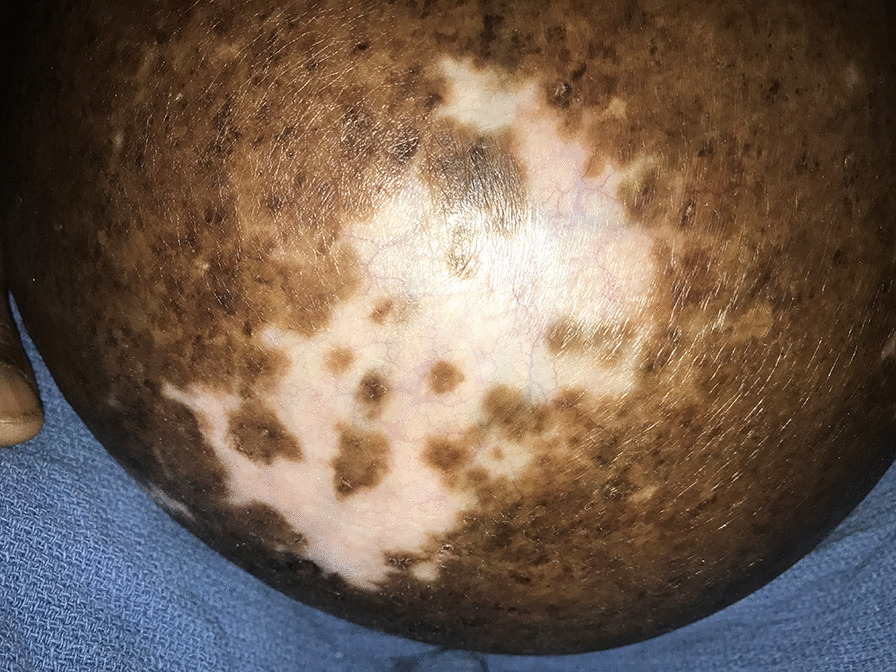
Healed ulcer on the anterior abdominal wall

On physical examination, the patient was febrile and tachycardic. Abdominal examination revealed an umbilical hernia about 25 cm × 20 cm with the anterior abdominal wall hanging down and extending downwards up to the mid-thigh (Fig. 1). A 32-week-size gravid uterus was palpable and the anterior abdominal wall was not reducible. The uterus and bowels were felt through the wall. There was also a well-healed skin ulcer on the anterior abdominal wall (Fig. 2).

Obstetric ultrasound showed gravid uterus herniated in the umbilical hernia leading to some limitations of the scan. Otherwise, the scan revealed a 31-week live single fetus in longitudinal lie with a fundal-anterior placenta, estimated fetal weight of 1800 g, and adequate fluid volume.

The patient was diagnosed with malaria, confirmed by malaria rapid diagnostic test, admitted to the antenatal ward, given lumefantrine-artemether (four tablets twice daily for 3 days) for malaria, and recovered very well. She was given dexamethasone for fetal lung maturation to mitigate the potential risk of respiratory distress if any preterm delivery occurred. After the patient recovered from the malaria, the pregnancy progressed uneventfully to term. The patient was counseled on the mode of delivery and contraception. She opted for a permanent method of contraception and noted that she preferred to undergo a scheduled surgery for cesarean, bilateral tubal ligation, and herniorrhaphy at 39 weeks. She preferred this plan to the alternatives of waiting for labor and ending up with a potential unscheduled cesarean and tubal ligation without appropriate staffing for herniorrhaphy, or having a vaginal delivery and then encounter delays with scheduling a postpartum tubal ligation with herniorrhaphy. Therefore, with the patient’s consent, caesarean section and bilateral tubal ligation, along with herniorrhaphy at the time of caesarean section, were performed at 39 weeks of gestation.

**Fig. 3:**
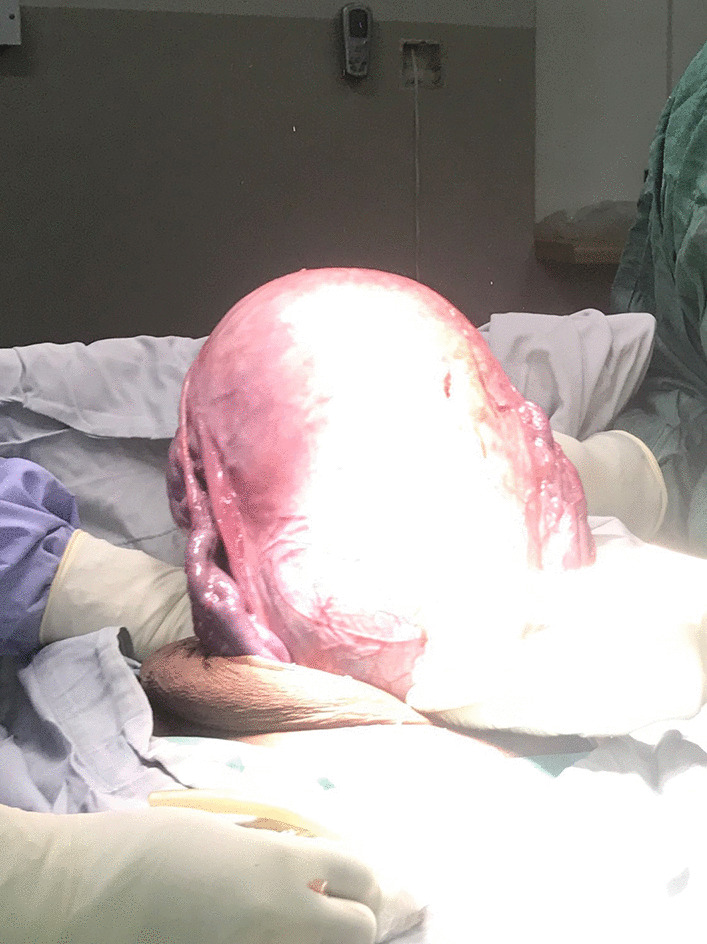
Exteriorized uterus prior to extraction of the baby

**Fig. 4:**
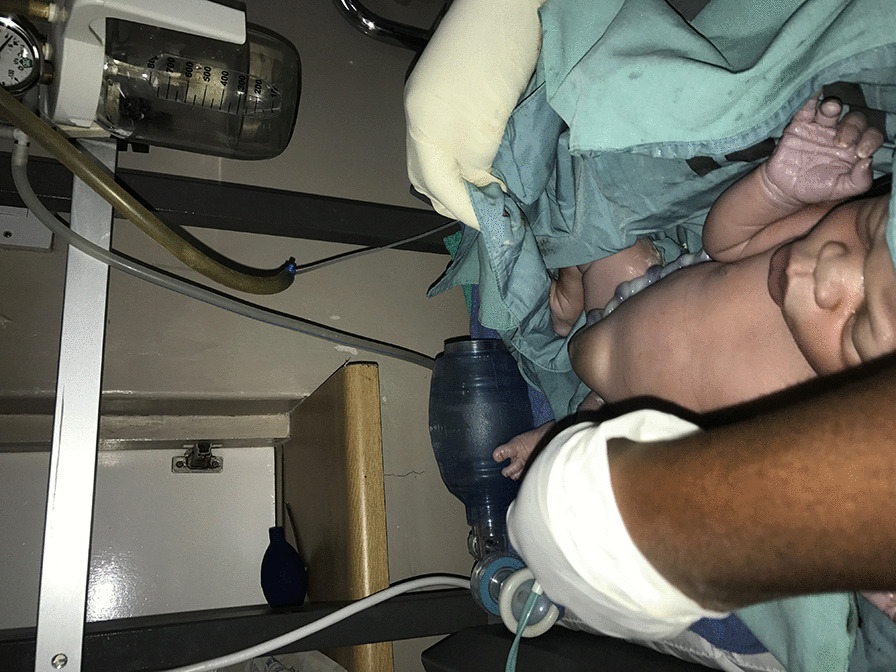
The newborn with congenital umbilical hernia

**Fig. 5:**
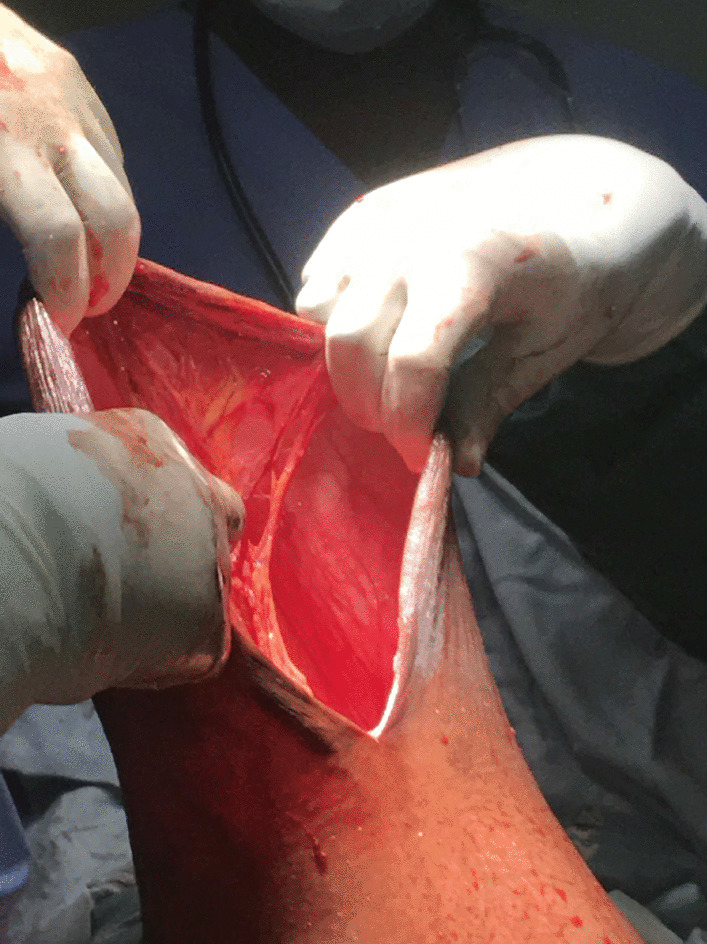
Excision of redundant skin

The abdomen was opened by supra-umbilical vertical incision. The hernia sac was incised; the gravid uterus was lying in the sac and was exteriorized prior to the delivery of the baby (Fig. 3). A transverse lower uterine segment incision was made, and we extracted a live full-term female infant weighing 3.7 kg with APGAR scores of 9 and 10 at 1 and 5 minutes, respectively. The infant was also noted to have an umbilical hernia at the time of birth (Fig. 4). After a bilateral tubal ligation was performed, the uterus was replaced in its normal pelvic position. Herniorrhaphy was performed with a running locking suture after excision of the hernia sac. Redundant skin and subcutaneous tissue were excised and the skin was sutured (Fig. 5).

The patient had an uneventful postoperative period and was discharged on day 4, and her stitches were removed on day 10. Our repair was still intact at the 3-month follow-up, with no evidence of herniation, and the patient was satisfied with the outcome. The infant was referred to the pediatricians for follow-up for the congenital umbilical hernia.

## Discussion

Umbilical hernia is common among African populations, with an estimated prevalence as high as 15% among pregnant women in West Africa [4]. Most umbilical hernias in adulthood are congenital and date back to childhood [5]. We reported a case of congenital umbilical hernia diagnosed first in pregnancy. Herniation of a gravid uterus in an umbilical hernia is rare because the gravid uterus is usually too large to enter the umbilical sac by the time it reaches the level of the hernia orifice [6].

There are sporadic reports of herniation of a gravid uterus through the anterior abdominal wall, and these are largely case reports. Most of the reported umbilical hernias in pregnancy were incisional hernias [7–10], and a few were congenital umbilical hernias [5, 11]. None of these cases reported an outcome of the baby also having a congenital umbilical hernia, as was the case with our patient.

Herniation during pregnancy in the umbilical sac may result in serious maternal and fetal complications. These include ulceration of the skin overlying the hernia, as was seen in our patient. Other reported obstetric complications that may result in maternal and neonatal morbidity include preterm labor, antepartum hemorrhage, rupture of the lower uterine segment, abnormal labor, postpartum hemorrhage, intrauterine growth restriction, and intrauterine fetal demise [6, 7].

Normal vaginal delivery has been accomplished in pregnant patients with the umbilical hernia and uterus lying within a hernia [3]. Our patient had three previous uneventful vaginal deliveries, with two of her babies weighing more than 4 kg each at birth. For our case, we opted for herniorrhaphy at the time of elective caesarean section, and we achieved a successful operative outcome. Controversy exists in the management of umbilical hernia in pregnancy due to the lack of literature describing the best evidence-based approach. Some authors recommend postpartum elective herniorrhaphy because the overstretched abdominal wall may interfere with wound repair and result in wound disruption or high infection rates [12]. However, others have performed herniorrhaphy at the time of caesarean section with a successful outcome, as in our case [9, 11, 13]. The use of mesh is another recommended option for repair of the hernia [11, 14]. We did not use a mesh for our patient because meshes are not available even when strongly indicated in our setting.

## Conclusion

Management of a patient with a gravid uterus in an umbilical hernia requires a patient-centered approach addressing patient complaints and possible complications. Knowledge of all possible complications and their unusual presentations can help achieve successful pregnancy and operative outcomes. Primary repair of an umbilical hernia at caesarean section is a feasible option.

## Data Availability

Not applicable.
